# Using a synthesis of the research literature related to the aetiology of adolescent idiopathic scoliosis to provide ideas on future directions for success

**DOI:** 10.1186/1748-7161-3-5

**Published:** 2008-02-02

**Authors:** Keith M Bagnall

**Affiliations:** 1 Department of Surgery, University of Alberta, Edmonton, Alberta, T6G 2H7 Canada

## Abstract

This review is atypical by design. It has used a synthesis of the available literature relating to the aetiology of AIS to draw attention to the lack of progress in this area despite intensive research for more than 100 years. The review has argued that if progress is to be made in this area then significant changes in approach to the problem must be made. Such changes have been outlined and major areas of potential focus identified with the intention of creating debate and discussion. There is no doubt that people are working hard in this area of research but this review has deliberately attempted to question its achievements and future directions.

"It is not enough to be busy, so are the ants. The question is what are you so busy about.'

Walden – Henry David Thoreau

## Introduction

This paper is not a typical review of the literature that might be expected when considering a review of adolescent idiopathic scoliosis (AIS). In this electronic age, using keywords such as 'scoliosis review' in Google or a medical database quickly reveals an enormous amount of material on this topic which should satisfy those people seeking fundamental, factual material (1478 'hits' by simply typing just 'scoliosis review' into PubMed database) [1-7]. Instead, this paper uses a synthesis of the available material to provide direction for future, more-productive and successful research.

Scoliosis has been recognized since at least the time of Hippocrates and presents itself as an abnormal lateral curvature of the spine accompanied by vertebral rotation. Interestingly, the plasticity of the elements of the spine allows scoliosis to take many different forms dependent on the multitude of different forces being applied to the various tissues making complex any classification and recognition of underlying causes. Some causes of scoliosis are readily apparent (e.g. congenital hemivertebra) and such cases have been removed from the general population of scoliosis patients because the underlying mechanism can be explained. Similarly, several diseases have been associated with scoliosis development (e.g. poliomyelitis, neurofibromatosis) and these too have been removed from the general pool of patients even though the underlying mechanism for the development of scoliosis is sometimes not clearly understood. However, even with the removal of these cases from the general pool of patients with scoliosis, there remains a large pool of patients (80%) who are justifiably classified as having idiopathic scoliosis because the cause remains unknown. Consequently, idiopathic scoliosis is a diagnosis by elimination rather than by positive identification which is a significant distinction.

Idiopathic scoliosis has three apparent peaks of time for diagnosis: congenital, infantile, and adolescent. AIS manifests itself at the time of puberty. Accordingly this paper focuses on AIS which is the most common type of scoliosis and which has received the most attention.

### Previous research into AIS

A simple review of the mountain of available literature shows that the aetiology of AIS has been explored extensively for more than 100 years. Seemingly, every conceivable aspect of development and tissue associated with the spine appears to have been placed under close scrutiny using the best techniques available at that time often with repeat evaluations occurring as techniques have improved or become available. Interestingly, the basic conclusions from this research can be synthesized as follows:

The development of AIS is associated with:

- *the growth spurt at the time of puberty*

- *greater severity of curve development in females than males*

- *multiple variations of deformity (single curves, double curves, curves to the left or right, different vertebral levels involved, different degrees of curvature)*

- *an inheritance within families*

Unfortunately, despite the intensity of the research over such a long period of time, these are the only reliable conclusions that can be made. When you consider the time and money that has been directed towards producing these few conclusions it is disappointing to realize that an observant visitor to a scoliosis clinic could reach the same conclusions probably within 20 minutes.

Why is this? Why have more significant advances into understanding the aetiology of AIS not been made? In contrast, there have certainly been significant advances made in the treatment strategies used for AIS and this area forms the bulk of presentations and materials at research meetings. The lack of parallel advancement in understanding the aetiology of AIS is disturbing and disappointing. It is further surprising because a better understanding of the aetiology of AIS would presumably result in the development of better treatment strategies but while there has been much interest, there has been little useful advancement.

With further thought, perhaps the four conclusions outlined above are not the only ones that could be drawn from the literature. Maybe another conclusion can be drawn:

*If major advances in understanding the aetiology of AIS are to be made, then the methods currently being used and the approaches being made must be changed because they have not been very productive so far*.

It has been said that one sign of madness is to continue doing the same thing repeatedly and expect different results. If the current methods to study the aetiology of AIS continue to be applied, why should different results be expected when such intense, detailed research in the past has revealed so little? What changes should be made if advancement in an understanding of the aetiology of AIS is to be achieved?

### Basic question to answer and its effects on experimental design

To study the aetiology of AIS efficiently and effectively, an experimental model needs to be developed to form the foundation of any experiments. The results from the experiments will either support the model as being correct or provide direction for making changes which can then be tested further. Fundamental to any model for the study of AIS is the question:

Do all cases of AIS have the same single underlying cause or are the abnormal curvatures the common end-result of several different causes?

While this is a very simple question, the answer has enormous consequences especially for experimental design and is an important question to be considered when viewing the available research literature.

If the answer selected on which to build an experimental model is that all cases of AIS have the same single underlying cause, then the traditional experimental design would be to obtain samples or measurements from a group of patients who have severe AIS (extreme cases) and compare the results to similar ones collected in the same way from a group of control subjects. The focus of the experiment would be on identifying any significant differences between the mean values of the measurements taken from each group. Experiments with this basic design are common in the literature but the results for any parameter being examined vary widely. It is no surprise that there is confusion and little forward progress being made when results from similar experiments are so diverse. Alternatively, perhaps there is a message in this important and extensive literature that has been overlooked. Perhaps the wide diversity and inconsistency in the results from similar experiments is suggesting strongly that the model being used (a single cause) might be wrong and that AIS actually has several different underlying causes (the alternative model).

If the true experimental model for AIS should be built on the premise of there being several different causes then no significant differences for any parameter would be expected using the traditional experimental design. Consider the following situation: if the hypothesis was that there were fewer spindles in the vertebral muscles of patients with AIS (hypothetical) then the traditional experimental design would include samples being collected from patients with AIS and appropriate controls. The samples would be sectioned and stained and the number of muscle spindles counted and compared between the experimental and control groups. Unfortunately, the mean values of the two groups of subjects would probably be very similar and not significantly different even though the hypothesis being examined (e.g. fewer muscle spindles) might well be an underlying cause of AIS. If fewer muscle spindles are the basic cause in only 10% of AIS patients, then their contribution to the mean values of numbers of muscle spindles in the group of AIS patients will be overwhelmed by the normal values from the 90% of AIS patients in the same group who have normal values but whose underlying cause is quite different. Unfortunately, this experimental design has been the used in many experiments performed in search of the aetiology of AIS and, not surprisingly, no reliable evidence has been found to support any conclusions for any parameter studied. In experiments designed in this way, the interesting values would be those of the 'outliers' in the experimental group rather than the mean values of the whole group but these extreme values rarely get reported.

If AIS has multiple, different causes then experiments designed with a single-cause being the underlying mechanism (such as described above) will not reveal any meaningful results even if the suspected cause of scoliosis being tested is quite valid. Reviewing the literature from this point of view suggests very strongly that the diverse results obtained so far confirm that AIS is the common end result of several different causes and not just a single cause common to all cases. Contributors to conferences and authors of manuscripts should be required to explain their experimental design to clarify this issue.

### Is there just one cause remaining to be found within the patients with AIS?

As groups of patients with associated diseases have been culled from the group of AIS patients, the associated disease has been recognized as the underlying cause. As these groups have been removed, the number of underlying causes within the remaining AIS group presumably has been reduced by one. This raises an interesting question:

In the current population of patients diagnosed with AIS, is there just one cause remaining to be identified or are there still several different causes within the population that are simply proving more difficult to identify?

For example, currently there is considerable discussion related to identification of syringomyelocoele in boys as being an underlying cause of scoliosis and debate exists over whether or not such cases should be removed from the general AIS pool. On this basis alone, it seems most likely that among patients currently diagnosed with AIS several separate causes have yet to be identified within the population rather than there being a single underlying cause common to all.

In contrast, the evidence to support AIS having just a single underlying cause is very limited. Certainly, curve progression seems to have many common characteristics and AIS can usually be recognized from a series of radiographs taken of a patient but there is not much else.

### Confounding issues affecting the research

#### Vacuous but critical time period when no measurements of patients with AIS are available

When a child develops AIS, they are unaware of its development in the early stages when the curve is small. Usually, it requires observation of sloping shoulders or other physical characteristic by another person before the lateral curve is recognized. By this time, the curve is already quite large for this manifestation to be noticed and has probably been present for several months, if not years, before a specialist is seen. This period of time when the abnormal spinal curves were first developing is critical for research purposes because the underlying cause will be present and yet it is a time of anonymity when no measurements can be made. Extrapolation of later measurements into this time period have proved to be limited because treatment strategies have affected curve development and, consequently, any extrapolation procedures.

#### The underlying cause might have gone at the time of examination and further curve development is purely a biomechanical problem

The biomechanics associated with spinal curve development are complex and not well understood. As it is usually several months (18?) from the time the curve started to when it is first recognized in a patient with AIS, it is possible that the underlying problem causing the development of the curve has been corrected without any treatment. The continued curve progression seen in AIS patients might then be due entirely to the biomechanical aspects of curve development rather than the continued effects of the underlying problem. In these cases, the patient with AIS might be completely normal when being examined! In particular, the experimental group in any experiment might well be perfectly normal in regards to the hypothesis being tested even though the hypothesis was in fact true – but only at the early stages of development. Presumably this would be particularly important when considering possible causes such as differences in hormonal production or timing of hormonal changes, parameters that are very evident during puberty and which might well have harmonized by the time of measurement. It is certainly a problem when patients with severe AIS are selected for the experimental group because, while such patients certainly represent the extreme cases, they are usually much older than when their curve first started to develop – which is the most important time for examination to identify the underlying cause. Unfortunately, the time of initial curve development cannot be identified.

#### Unable to separate cause from effect

At present, patients with AIS cannot be identified prior to initial development of the spinal curve. There is no marker available which can predict future scoliosis development. Consequently, it is almost impossible to separate cause from effect of the scoliosis from any measurement taken from patients with AIS because there are no earlier measurements available for comparison. This makes interpretation of results from experiments involving AIS patients very difficult to interpret as a genuine cause of scoliosis with any degree of confidence when considering the experimental parameter because any significant changes found could be the result of the scoliosis.

#### Necessity of bipedal stance ensures no appropriate animal model can be identified

A bipedal stance appears to be necessary for development of scoliosis and Man is the only true bipedal animal. Bipedalism arose in Man because the increased lumbosacral angle allowed Man to hold his head and torso high while retaining quadrupedal characteristics in the pelvis and lower limbs, necessary for locomotion. No other animal appears to have adopted the same evolutionary pattern. Consequently, the selection of an animal on which to develop a research model is very difficult. Avians, with their pseudo-bipedal stance, have been used with some success but there is a large phylogenetic gulf between birds and mammals and significant differences in physiology and biochemistry make progress in understanding any mechanism very difficult. For example, pinealectomy in newly-hatched chickens often results in scoliosis development but similar surgery in young mammals (quadrupeds) has no effect UNLESS they are also made bipedal by removal of their upper limbs and tail. This model certainly has potential but questions remain concerning the effects associated with the extent of deviation from normal morphology as well as actual time spent in a bipedal stance when compared to normal, quadrupedal mammals.

#### Multifactorial and multi-elemental

A strong case has been made in this review to suggest that among the population of patients with AIS there are several, different underlying causes that remain to be identified. Consequently, the cause of AIS is described as being multifactorial. Unfortunately, the problem is probably much more complex than simply being a series of different underlying causes because each separate cause might consist of several contributing elements themselves. For any single underlying cause, these constituent elements might also not be equal in importance with some elements being essential for future curve development while others may or may not need to be present either alone or in combination with the other elements for any spinal curve to develop. This interweaving of the threads associated with scoliosis development creates a complex knot and, currently, there is insufficient data to create a theoretical model on which to build.

### Areas of research that appear to have the potential for the greatest success in the near future

#### Detailed re-read of the literature

Despite the criticism of the available literature outlined above, there remains a wealth of information contained in the literature which could be gained by re-reading the literature using a different approach. The useful data is mixed in with data from badly designed experiments which have been designed based on a model of AIS having a single underlying cause (as described above). Such experiments need to be identified and their data re-evaluated and either removed or incorporated into a new database of information. In particular, the outliers from experiments designed with a single cause as the model should be identified if possible and their values looked at very carefully.

#### Development of an idea related to the underlying cause of scoliosis

If many underlying causes for AIS remain to be identified, then they need to be evaluated and explored separately. However, this cannot be approached in the traditional way of collecting samples and measurements from a group of patients with AIS and comparing these values with similar measurements from a control group. Instead, a new approach needs to be developed along the lines of:

- develop a theory based on the literature and thoughtful ideas

- consider if it can be demonstrated in an appropriate animal model

- outline the symptoms that would be seen in a specific group of patients with scoliosis if the theory was correct

- search for such patients retroactively or among current patients

This approach is far more laborious (needle in a haystack?) than the traditional approach but any results would be positive and constructive.

#### Genetics

There are two types of molecular approach that would be productive:

#### Mendelian genetics

It is well recognized that there is a familial relationship in scoliosis. However, this relationship is confusing and not well defined probably because of the complex nature of AIS, especially the multifactorial (more than one cause) and multi-elemental nature of the underlying cause. The environmental influence on scoliosis development is also unknown but the fact that there is only 70% concordance in identical twins diagnosed with AIS illustrates its significance. Similarly, it is interesting to note that the concordance between dizygotic twins (basically, siblings in the same uterus) is only 37% and yet the concordance between non-twin siblings is only 6–7%. Such differences raise the question of what influence does prenatal environment have on scoliosis development? The data to explain these differences and the influence of the environment on scoliosis development needs to be expanded so that the focus of research can be more directed.

#### Molecular genetics

Currently, identifying genes that are different to normal in patients with a specific disease is receiving major attention in many areas other than AIS. In some instances, single specific genes have been identified and make for dramatic headlines. Other diseases have been found to be much more complex with several, if not many, genes being identified as being involved with the disease. AIS falls into the latter group of diseases even though there have been headlines claiming the finding of just single genes being identified as the underlying cause of scoliosis. Again, the problem would probably be reduced if it was possible to identify curves that had developed from different causes and study them separately but, currently, the pool of AIS patients is heterogeneous with a mix of all types of underlying cause in most cases. Certainly, more-refined strategies where members from the same family are being examined will probably reduce the number of suspect genes but if the underlying, single cause has many constituent elements then the situation and model to be developed becomes very complicated and continues to be difficult to solve. Adding to the complexity, the concordance for AIS between monozygotic twins is only 70% which implies that there are some people with identical DNA where one has developed scoliosis while the other has not. Furthermore, how do you identify the contributing genes for scoliosis among family members where the DNA is not identical when scoliosis may or may not develop among people with identical DNA! Similarly, if the complexity of the multifactorial, multi-elemental model proposed for the aetiology of AIS in this review is accepted, then it is quite possible that the lack of concert in gene expression for the elements within a single cause means that some members of the same family will possess similar critical genes but because of the differences in timing patterns, some members of the family develop scoliosis while others do not. This will be a long and difficult process [8].

It used to be thought that 'one gene' meant 'one protein (or enzyme)' but recent advances in cell biology have revealed that gene expression is modified at various levels before protein expression occurs with, in at least one case, at least 180 proteins being found to be expressed from one gene. The unexpected, relatively few genes found in the human genome appears to have been a mask to hide the real complexity and volume of proteins being expressed.

Nevertheless, given the success of molecular genetic research in making progress in understanding the aetiology of other diseases, a molecular genetic approach to the aetiology of AIS appears to have the best chance for long-term success. Perhaps it is also possible to make even more rapid progress by copying the methods employed in research of a disease with similar characteristics to AIS but it is difficult to identify one which might be appropriate for comparison because of its complexity. Suggestions for a comparative disease might include cancer and autism, both of which appear to have similar end-results among their patients but which have apparently many different underlying causes. However, it must be realized that even though the genetic differences from normal have been recognized for many years in many diseases, very little progress has been made in introducing corrective genes to the patient.

#### Revelation of the path between cause and curve development

Even if an association is made between scoliosis and a particular characteristic (e.g. poliomyelitis, cerebral palsy) there is a long path and much explanation required to connect the underlying mechanism with the development of a spinal curve. What is perceived as an association between a particular characteristic and AIS must be connected to a mechanism to produce the spinal curve – and this is often a very difficult and complex path to understand and isolate. Nevertheless, research in this area would be valuable because it would provide insight into the mechanisms of scoliosis development which is currently poorly understood and guide development of successful treatment strategies [9,10].

### Engineering

There are three prime areas in which a prominent engineering approach would be beneficial and valuable in obtaining a better understanding of the aetiology of AIS:

### Understanding 3-Dimensional (3D) curve structure and performance

Postero-anterior and lateral radiographs have been used routinely to observe overall curve morphology, especially degree of curvature, as well as curve development between clinic visits. Such images allow a basic 3D image to be created in a rather primitive fashion but it has proved particularly useful to the surgeon in deciding treatment. However, it must be remembered that a radiograph is compression of a 3D structure into 2D and has many inherent problems for interpretation. Scoliosis is a 3D deformity and, as such, should always be thought of in 3D terms. In particular, the compression of a 3D structure into 2D (as in radiographs) does not allow for the exclusion of the same spinal curve being thought of as changing in severity and classification between visits when only simple rotation of the spine has occurred. The ability to observe or image the spine in 3D has improved enormously in recent years and its availability has also increased especially with refined development of MRI technology. If a complete understanding of spinal curve development is to be achieved, then research evaluations have to move away from 2D imaging such as with basic radiographs and observe only 3D images. 2D assessment should be no longer acceptable for research purposes and new techniques of 3D assessment need to be developed to keep pace with the improvements in imaging techniques. The following statement highlights this area:

*'Measure in 2D, you think in 2D; measure in 3D, you think in 3D' – and scoliosis is a 3D problem*.

Visits to the scoliosis clinic by patients provide the opportunity to see 'snapshots' along the continuum of spinal curve development. However, spinal curve development is a continuous process and not simply a series of images separated by time. The development of techniques to produce a continuous image (movie?) of curve development from the images collected from patients during visits to the scoliosis clinic would be very valuable. Just as more information would be available by observing curve morphology using 3D imaging, even more information would be available if continuous imaging of curve development was also introduced. There is an opportunity for such techniques to be developed because technology has caught up with the desires of researchers – it is simply a matter of defining the techniques wanting to be created.

### Biomechanical involvement in curve development and treatment

As a spinal curve develops and increases, it seems reasonable to imagine that the biomechanics associated with column construction affect curve development and associated rotation and that their influence increases with curve progression. It also seems reasonable to suggest that while the abnormal spinal curve remains small, removal of the underlying cause would allow the body possibly to return the curve to normal. An understanding of the biomechanical principles involved with column structure and curve development would be invaluable to understanding the aetiology of AIS but is currently unavailable. Extending this concept, it also appears possible that a point of abnormal curve development might be reached during progression at which correction cannot be achieved by the body even if the underlying cause was removed. Such a point might be related to morphological changes such as those seen with vertebral wedging but the realization that curve development will continue to progress even with the underlying, deforming force removed would be valuable in the development of treatment strategies especially in the area of providing supportive treatments such as those associated with 'bracing' [11].

### Development of computer models

Perhaps the ultimate research tool would be the development of computer models to simulate spine morphology and normal growth which could include curve development. Such techniques have had outstanding success in other areas of research and there are certainly seeds of development of these techniques in scoliosis. Clearly, the potential for unparalleled success in understanding the mechanics of scoliosis using such techniques suggests strongly that this area of research should receive much more attention in the near future.

#### Four basic questions that should be the focus of attention in any research

The lack of any really substantial knowledge relating to the aetiology of AIS as seen in the literature emphasizes the basic areas that should form the focus of all current research because knowledge in these areas is most critical and readily applicable:

*The development of a marker to identify those children who are going to develop AIS. This would allow treatment strategies to be developed to avoid even initial curve development*.

*The development of a marker which would indicate whether a small, abnormal spinal curve will progress, remain the same, or regress. This would allow aggressive treatment strategies to be applied to those patients whose small curves have been identified as going to progress*.

*The development of techniques to treat small, spinal curves that have already developed to ensure that they do not progress and are corrected instead*.

*The development of new techniques to replace the extensive and invasive techniques currently being used (successfully) to repair large, abnormal spinal curves*.

## Summary

In his recent successful book 'Where have all the leaders gone?' Lee Iacocca, the former CEO of Chrysler, reports coming into contact for the first time with medical researchers (for diabetes) in his retirement years. He commented that medical research was much like government – kind of a self-generating bureaucracy. Cynically, he noted that people do research so that they can write papers to get more funding to do more research to write more papers. He was prompted to ask: 'Hey, isn't anyone trying to find a *cure*?' The goal for research into the aetiology of AIS should be to find a cure (other than surgery) as soon as possible, preferably tomorrow – but, unfortunately, a cure remains a dream and does not even seem to be on the distant horizon. Researchers in this field, particularly basic science researchers, should ask themselves if their work has produced results that have affected the treatment of even a single patient with AIS and, if not, then why not?

*Finally, a plea for better communication: *in today's electronic and computerized environment with relatively easy access to video cameras and the ability to both see and hear each other so readily available, could a world-wide research environment be created that involves all interested researchers in all the different areas with regular intercourse and discussion?

## References

[B1] Anderson SM (2007). Spinal curves and scoliosis. Radiol Technol.

[B2] Asher M, Burton D (2006). Adolescent idiopathic scoliosis: natural history and long term treatment effects. Scoliosis.

[B3] Cheung KM, Wang T, Qiu GX, Luk KD (2007). Recent advances in the aetiology of adolescent idiopathic scoliosis. Int Orthop.

[B4] Everett CR, Patel RK (2007). A systematic literature review of nonsurgical treatment in adult scoliosis. Spine.

[B5] Goldberg CJ, Moore DP, Fogarty EE, Dowling FE (2007). Scoliosis: a review. Pediatr Surg Int.

[B6] Robin G (1990). The etiology of idiopathic scoliosis: a review of a century of research.

[B7] Scoliosis Research Society. http://www.srs.org/patients/review/default.asp?page=2.

[B8] Miller NH (2007). Genetics of familial idiopathic scoliosis. Clin Orthop Relat Res.

[B9] Grivas T, Savvidou O (2007). Melatonin the "light of night" in human biology and adolescent idiopathic scoliosis. Scoliosis.

[B10] Hawes M, O'Brien J (2006). The transformation of spinal curvature into spinal deformity: pathological processes and implications for treatment. Scoliosis.

[B11] Stokes I, Burwell G, Dangerfield P (2006). Biomechanical spinal growth modulation and progressive adolescent scoliosis – a test of the 'vicious cycle' pathogenetic hypothesis: Summary of an electronic focus group debate of the IBSE. Scoliosis.

